# Key ingredients for successful collaboration in health research: perspectives of patient research partners

**DOI:** 10.1186/s40900-024-00590-2

**Published:** 2024-06-05

**Authors:** Marcia Bruce, Karthika Yogaratnam, Nitya Suryaprakash, Karis L. Barker, Deborah A. Marshall

**Affiliations:** 1 Department of Medicine, Cumming School of Medicine, University of Calgary, 3280 Hospital Drive NW, Health Research Innovation Centre (HRIC) Building, Room 3C58, Calgary, AB T2N 1N4 Canada; 2 Department of Community Health Science, University of Calgary, 3280 Hospital Drive NW, Calgary, AB T2N 1N4 Canada

**Keywords:** Patient-oriented research, Patient engagement, Patient involvement, Qualitative methodology, Patient experiences

## Abstract

**Background:**

There are increasing publications on meaningful collaboration between researchers and patient research partners (PRPs), but fewer publications of such work from the PRP perspective using an evaluation framework. Our aim is to present our own perspectives and reflections on meaningful collaboration as PRPs working on a qualitative research study.

**Main body:**

We were part of a study team that comprised of PRPs, clinicians and academic researchers, and was led by a PRP. The team designed and conducted a qualitative study aimed at understanding how patients make decisions around tapering of biologics for inflammatory bowel disease. The study was conducted online. The PRP lead was trained in qualitative methodology through a one-year certificate program called Patient and Community Engagement Research offered through the University of Calgary Continuing Education. We had received patient-oriented research training and qualitative research training prior to this project. Team members were assigned tasks by our group lead based on member interests and willingness. Some group members were part of the Strategy for Patient-Oriented Research, Inflammation, Microbiome, and Alimentation: Gastro-Intestinal and Neuropsychiatric Effects Network, one of five chronic disease networks in the Strategy for Patient Oriented Research initiative of the Canadian Institutes of Health Research. We describe the five key ingredients to successful collaboration based on our experiences and reflections utilizing the Experience-Reflection-Action Cycle as our framework. The five key ingredients that we identified were: inclusiveness, goal and role clarity, multi-level training and capacity building, shared decision making, and a supportive team lead.

**Conclusion:**

Overall, our experience was positive. With successful collaboration came an increased level of trust, commitment and performance. There is a need for more studies with diverse PRPs in different settings to validate and/or identify additional factors to improve collaboration in patient-oriented research.

## Abstract (plain language summary)

There are an increasing number of publications on collaborative research work between researchers and patient research partners but fewer publications of such work from the patient research partner perspective. We share our experiences, reflections and the key factors for successful collaboration as two patient research partners on a research team led by a patient research partner trained in qualitative methodology through a one-year certificate program called Patient and Community Engagement Research offered through the University of Calgary Continuing Education. We had a positive overall experience and contributed as equal partners on the project, influencing many of the project decisions. Inclusiveness, goal and role clarity, multi-level training and capacity building, shared decision making, and a supportive team lead contributed to the feeling of empowerment and satisfaction. Our results indicate that with successful collaboration comes an increased level of trust, commitment and performance. There is a need for more studies with diverse patient research partners in different settings to validate and/or identify additional factors to improve collaboration in patient-oriented research.

## Background

Including patients in health research can help to ensure that research activities, and the research findings and recommendations that are generated, are better aligned with the needs of the patient community [1]. The Canadian Institutes of Health Research (CIHR) describes patient engagement as meaningful and active collaboration in governance, priority setting, conducting research and knowledge translation [2]. Patient research partners (PRPs) can: (i) be involved as advisors on certain aspects of the project; (ii) take on operational roles and conduct some parts of the research; and, (iii) lead or co-lead projects.

**Fig. 1:**
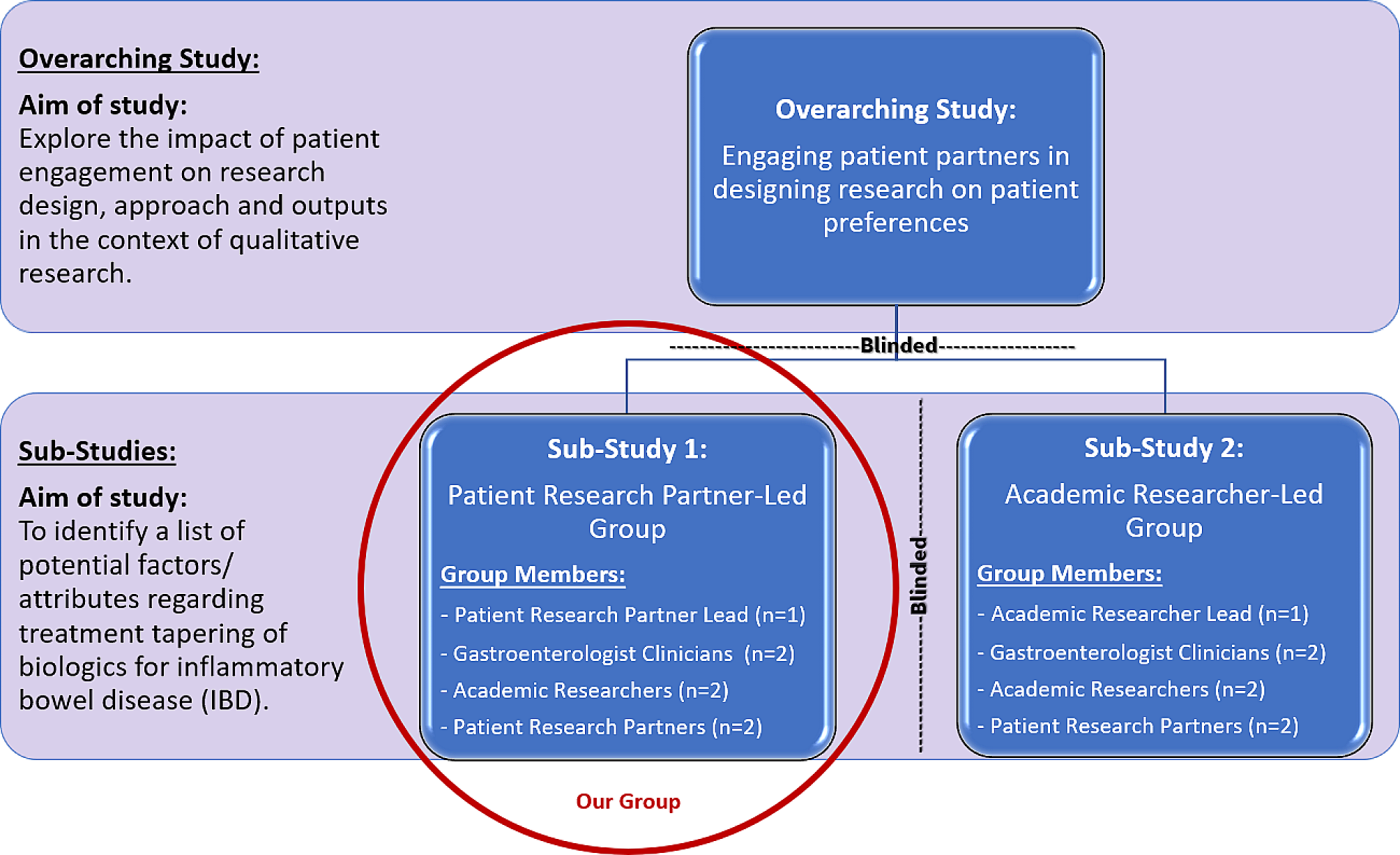
Study design

We, the first two authors of this paper, had a unique opportunity to collaborate as patient research partners in a qualitative research study aimed at understanding how patients make decisions about tapering of biologics for inflammatory bowel disease (IBD). This study was part of an overarching investigation aimed at exploring the impact of patient engagement on research design, approach and outputs in the context of qualitative research [3–5]. The research team for the overarching investigation formed two distinct research groups to study patient engagement, one group that was led by a PRP and one that was led by an academic researcher (see Fig. 1) - we were part of the PRP-led group.

Our seven-member group was comprised of three PRPs, two gastroenterologists, and two academic researchers. The PRP who led our group was trained in qualitative methodology through a one-year certificate program called Patient and Community Engagement Research (PaCER) offered through the University of Calgary Continuing Education [6, 7]. Both PRP authors had completed an internship-style qualitative research project as part of our training and education, with one of us being trained under the PaCER program and the other having qualitative research training through her master’s degree.

All team members were familiar with IBD to some degree, whether through lived experience, previous IBD research knowledge, or through providing clinical care to patients with IBD. Some team members were part of the Inflammation, Microbiome, and Alimentation: Gastro-Intestinal and Neuropsychiatric Effects (IMAGINE) Network, one of five chronic disease networks in the Strategy for Patient Oriented Research (SPOR) initiative of CIHR [8].

Our aim is to present our own perspectives and reflections on meaningful collaboration as PRPs working on a qualitative research study.

## Main body

According to Riches et al. [9], including people with lived experience on project teams is foundational and important throughout the research cycle. But often, as PRPs on research teams, we are left feeling that our expertise as people with lived experience, and our other skills and professional expertise that we bring to the team is undervalued. This was the first research team where we felt that we were experts in our own way and that we were able to meaningfully contribute throughout the entire research cycle. In this paper, we use the Experience-Reflection-Action (ERA) framework [10] to identify the key factors, or ingredients that contributed to our positive experience and satisfaction. The ERA framework is a simple, straightforward model grounded in reflexive actions where one:

1. Considers their experiences (whether good or bad),
2. Reflects on lessons learned from those experiences, and
3. Implements changes or actions based on those learnings.

Reflecting on our experiences on this patient-led project, we identified five key ingredients that contributed to our successful collaboration: inclusiveness, goal and role clarity, multi-level training and capacity building, shared decision making, and a supportive team lead. Refer to Table 1 for a summary of these key ingredients.

**Table 1:** Five key ingredients for successful collaboration using the ERA Framework

Category	Our Experiences (what we did)	Our Reflections (our feelings and thoughts based on the experience and how we were impacted)	Our Recommendations (actions that might help and what could be done differently)
**Inclusiveness**	The team had the right mix of patients, researchers and clinicians, with all three stakeholder groups equally represented. Team members had diverse backgrounds and brought their own set of skills and knowledge to contribute to the team, and accomplish the study objectives. We adopted a broad perspective when sharing our opinions during discussions and tried to include ideas that we heard over time.	We really appreciated having other PRPs on the team as it was less intimidating than when you are the only patient on a team full of academics and clinicians. We were both experienced PRPs and had research knowledge and skills to collaborate. We wondered if the experience on the team would have been the same for PRPs who were not as knowledgeable about research practices.	It would be helpful for research teams to consider including at least two patient research partners with diverse backgrounds and experiences on research teams Researchers could recruit a mix of new and more experienced patient partners to bring in fresh perspective and aid in capacity building. PRPs should be encouraged to discuss broad concerns of interest that would be representative of others, not just themselves.
**Goal and role clarity**	All team members discussed the project goal and research question prior to start of the project. There was confusion about the research question. The team worked together to finalize a concrete question. All team members discussed personal goals, how they would like to engage on the project, and time commitment prior to the start of the project. The team leader divided the work according to individual strengths, willingness and time availability of team members. We took on responsibilities and executed tasks with confidence once we agreed to our roles.	Discussions about the research goal and question helped us understand what the project was about. We felt motivated to be involved and think about patient-centric ways to achieve the goal. It was easier for us to operate with a set goal and plan. Dividing the project work was an efficient way to move the project forward within the given timeframe. There were no misunderstandings as we discussed our expectations and roles upfront. We liked that our own personal project goals were discussed and our time commitments were taken into consideration before the start of the project work. We felt a shared sense of purpose and commitment to the collective goal.	There should be transparency and clarity regarding the purpose of the study, goals and roles. Roles should be defined in conversations with each PRP about their skills, interests, and willingness to contribute. No role should be undervalued or overlooked when it comes to collaborative research. Full disclosure of time commitment and availability is required for successful collaboration. As the project progresses new interests or challenges may arise, so there should be regular check-ins with PRPs to see if roles need to be adjusted.
**Multi-level training and capacity building**	We both had POR training and had completed an internship-style qualitative research project as part of our training and education. We were provided minimal formal training: a video on patient preference and qualitative research. The researchers and clinicians on the team received the same video. There was a wealth of opportunities to learn new skills from each other and gain valuable insights. Informal training happened during the meetings (e.g. NVivo software overview).	Our qualitative and patient-oriented research training gave us credibility and prepared us to be productive and competent contributors to all aspects of the project from the start. We think that the researchers felt confident in our ability to execute our tasks. Our training and experience in patient-oriented research also helped us understand the different roles, biases, and challenges that could potentially generate conflict or distrust.	PRPs should be provided training on research cycles and relevant methodologies. Training can be either more or less intensive depending on the willingness, interest, and roles of PRPs on a project. Training could be provided prior, and/or ongoing throughout the project. Academic researchers and other team members should also receive training on engaging and working with PRPs.
**Shared decision making**	We did not have a lot of time to get to know each other. We were a part of the decision-making process from the preparatory phase to the knowledge translation phase of the study. There was two-way communication. We faced scheduling challenges since team members were living in different parts of Canada and had competing priorities. The team discussed mechanisms for continuous communication and feedback and came to a consensus to use multiple modes such as OneDrive to share documents and weekly videoconference team meetings. We proposed a study design and approach that was patient-centric without compromising the scientific rigor of the study. We were heavily involved in creating patient-centric focus group and interview guides, and subsequently conducting the focus groups and interviews independently with patient participants.	We did not feel insecure or uncomfortable. There was mutual respect. We felt like equal members of the group. It did not feel as though there was a formal hierarchy. We felt trusted and valued, like our opinions mattered at every stage of the project, which was critical to our engagement and commitment. Having different ways to communicate, especially asynchronously, became a strength of the team as it created flexibility by accommodating different communication styles. There was transparency in real-time. The team worked cohesively, understanding problems and finding the solutions. Responsive dialogues allowed for active participation in the co-learning process with a broad range of perspectives and expertise utilized in guiding the project’s direction. Individually, we both were satisfied and motivated.	Projects should include more time and, if possible, flexible timelines so that teams can build a trust-based relationship. There should be shared leadership and decision-making processes at all levels and phases of the research. Inclusive mechanisms and processes should be set up at the start of the project. PRPs should be provided opportunities to co-lead projects with researchers.
**Supportive team lead**	The team lead promoted interactions between team members and ensured every team member contributed to the discussions and decisions made during meetings. If a team member was not able to attend the meetings, they were informed through weekly update emails. The team lead provided the same resources to all members regardless of whether they were PRPs, clinicians or researchers. All team members participated in a reflexivity exercise to discuss potential unconscious biases and conflicts of interest.	We felt we were in a safe and inclusive environment that bolstered our contributions to the discussions and decisions. We spoke our mind when we had concerns about the research design, approach or any of the study documents during all project phases.	It is important that the team lead create a diverse and inclusive environment so that all team members can feel welcomed, have honest conversations and continue participation on the project. Study team leads should have excellent communication, organizational, and relationship building skills. Someone who actively listens to each team member, asks for advice, ideas and feedback. They should also be able to manage meetings effectively.

1) Inclusiveness

It is often challenging as PRPs to share our unique perspectives, especially at the beginning of a project, and it can be even more challenging if you are the only PRP on the team. On this project, we had the right mix and number of patients, researchers and clinicians to accomplish the study objective. We really appreciated having other PRPs on the team as it was less intimidating than when you are the only patient on a team full of academics and clinicians.

We were both experienced PRPs and had research knowledge and collaboration skills. We adopted a broad patient perspective when sharing our opinions during the discussions and tried to include ideas from other patients that we have interacted with to share more than just our own opinions. We wonder if the experience would have been the same for PRPs who are not as well trained or as knowledgeable about research practices as us.

It would be helpful for teams to consider having representation of at least two patient research partners with diverse backgrounds and experiences on research project teams. This number has been recommended by other researchers [11, 12] and also takes into consideration attrition over the course of the project due to health reasons or personal reasons such as travel. Martineau et al. suggest that projects include a mix of new and more experienced patient partners to bring in fresh perspective and aid in capacity building [13].

2) Goal and role clarity

Role clarity is another key ingredient to the success of a collaborative team [14, 15]. There should be transparency and clarity on the purpose of the study, the work that will be required and full disclosure about the time commitment and availability. Our group leader had a vision, a clear plan for the project, and set us up for success by providing sufficient opportunities for all team members to discuss our goals, and how the team was going to achieve the project objectives.

All team members discussed the project goal and research question at the beginning of the study. There was some confusion about the research question, but the team was able to use our collective knowledge to help finalize a concrete question. All team members also discussed their personal goals, how they would like to engage on the project as well as time commitments and availability. The team leader divided the work according to the individual strengths, willingness and availability of team members. There were no unreasonable demands on any group members, and everyone seemed happy with their roles.

We liked that our own personal project goals were discussed and our time commitments were taken into consideration before the start of the project work. We felt a shared sense of purpose and commitment to the collective goal. We were happy and clear about our role on this project. Discussions about the research goal and question helped us understand what the project was about and gave us an opportunity to think about patient-centric ways to achieve the goal. It was easier for us to operate with a set goal and plan. Dividing the project work was an efficient way for moving the project forward within the given timeframe.

As PRPs continue to be involved in health research, research teams must try to make sure that more people understand the role of PRPs on research teams so that patient research partners can achieve positive, meaningful experiences. Reflecting on our experiences with this project, we recommend that roles for PRPs should be defined in conversations with each PRP about their skills, interests, availability, and willingness to contribute. No role should be undervalued or overlooked when it comes to collaborative research. Also, as the project progresses and PRPs are exposed to other areas of the project, new interests or challenges may arise, so there should be regular check-ins to see if roles need to be adjusted.

3) Multi-level training and capacity building

When patients join research teams without proper training and experience there is a risk of tokenism. Tokenism [1, 11, 16] is a concept that many patients who have been involved in health research for a while are familiar with. As PRPs, we have both experienced tokenism; where our responsibilities and roles on the team were reduced compared to the roles for which we were recruited. That did not happen with this team, we felt like our opinions mattered throughout the project. We took on roles and responsibilities that exceeded our initial expectations, which was critical to our engagement and commitment. We were provided minimal formal training, however, we had many opportunities for informal training and learning during our frequent team meetings discussing various aspects of the project, such as an overview of NVivo software and strategies for analysis.

Our previous training prepared us to be productive and effective contributors to all aspects of the project and also provided knowledge exchange opportunities between members of the group because we were familiar with the research cycle, methodologies and terminology used during the discussions throughout the project. Our training and experience in patient-oriented research also helped us understand the different roles, biases, and challenges that could arise and create conflict or an environment of distrust that would be detrimental to the success of our collaboration. We also think that the researchers felt confident in our ability to execute and accomplish our tasks.

To help them prepare, and have confidence in their roles, PRPs should be provided training on research cycles and relevant methodologies, especially if they do not have prior training. Training can be either comprehensive, like the University of Calgary’s PaCER program [6], or less intensive like the Partners in Research (PiR) 2-month online course run by the IMAGINE Network [17], depending on the willingness, interest, and roles of PRPs. Preferably the training would be completed prior to a study commencing, but some research teams might not have enough resources (money and time) to train PRPs at the preparatory phases of projects. If PRPs cannot be trained before the study begins, training opportunities should be made available as soon as possible, and accessible throughout the duration of the project. The training provided should be based on the roles that the PRPs will play on the team and the skills that will be required for them to be successful (e.g., software training, or training in the relevant methodologies). Academic researchers should also receive training on engaging and working with PRPs.

We would appreciate it if researchers who may not understand the full scope of patient-engaged research turn to trained PRPs to guide them or to organizations such as the CIHR Strategy for Patient Oriented Research [18], Patient-Centered Outcomes Research Institute [19] and National Institute for Health and Care Research [20] who all have mandates to improve patient partnership in health research.

4) Shared decision making

Collaborative work normally requires a level of personal familiarity, intimacy and trust. Our team worked under tight deadlines resulting in insufficient time for getting to know each other. However, this did not significantly hinder how we worked together because there was shared decision making at all levels and phases of the research that helped build trust over time. Unlike some of our previous experiences as PRPs, where our roles were limited to sharing our lived experience and helping with recruitment, in this project, we were a part of all of the decisions that were made. We took on responsibilities and executed our tasks with confidence once we agreed to our roles. We proposed a study design and approach that was patient-centric and included a range of diverse patients without compromising the scientific rigor of the study. We were heavily involved in creating patient-centric focus group and interview guides, and subsequently conducting the focus groups and interviews with patient participants. Once the data were collected and analyzed, we discussed the results of the literature review, focus groups and interviews, and we finalized the key findings with our other team members.

Shared decision making led to our team working cohesively. We felt like equal members of the group. We felt trusted and valued, like our opinions mattered at every stage of the project. We wanted to ensure quality results were delivered to help validate that trained patients can contribute broadly to all aspects of the research process, not just in select project activities. In their study, Leese et al. found that patients partners valued environments where they were heard and their contributions were considered equally important as the rest of the team [21].

Our team faced a challenge with scheduling since team members were collaborating from different time zones and had competing priorities that limited the ability to meet in real-time. To overcome our scheduling challenges, all team interactions were virtually-based using videoconferencing or asynchronous using collaboration software where we left comments and questions for each other in shared documents. Even though we did not have a lot of time to get to know each other and prepare for the undertaking, everyone made time to discuss ways to communicate and acknowledge the different communication preferences of members to maximize the team effectiveness. Having different ways to communicate, especially asynchronously, became a strength of the team as it created flexibility by accommodating different communication styles. It also created transparency in real-time.

Reflecting on this experience, we recommend shared decision making processes at all levels and phases of the research. Teams should set up inclusive mechanisms and processes at the start of the project for successful collaboration.

We also propose that PRPs be provided with increased opportunities to co-lead projects with researchers. We think this strategy may reduce the power differentials between patients and researchers, and lead to more meaningful engagements with patient groups. Lauzon‑Schnittka et al.’s review paper [22] noted that when hierarchies are abolished, PRPs feel empowered to contribute, leading to more positive experience and satisfaction working on projects.

5) Supportive team lead

The leadership skills demonstrated on this project played a big role in the success of our group. For us as PRPs, having a group leader who was also a PRP made her more approachable and changed the working dynamics since we saw her as a peer. It did not feel as though there was a formal hierarchy, which contributed to everyone pitching in when their expertise was required. It felt easier to be vulnerable and speak our minds, which empowered us and increased our level of engagement throughout the study.

The team lead promoted interactions between team members and ensured that every team member contributed to the discussions and decisions. Our leader spent a lot of time outside of group meetings preparing materials and drafting documents so that when we were able to meet as a team, the time together was productive and used to have generative discussions resulting in shared decision-making to move the work forward. If the member was not able to attend the meetings, they were informed through weekly update emails.

The team lead also provided the same resources to all members regardless of whether they were PRPs, clinicians or researchers. All team members participated in a reflexivity exercise to discuss potential unconscious biases and conflicts of interest.

The safe and inclusive environment created by our team leader bolstered our contributions to discussions and decisions. We spoke our mind when we had concerns about the research design, approach, or any of the study documents during all project phases.

Some key qualities that leaders should have include relationship building to understand what roles the PRPs on their team would like to play, organization and the ability to manage meetings effectively to allow a safe space for patients to contribute, and excellent communication skills to ensure everyone on the project team is up to speed.

## Conclusion

Patients can and should be embedded within health research teams to ensure that the research is aligned to meet the needs of the patients who are impacted by the research. With appropriate training in research methodology, patient research partners can be empowered to contribute to multidisciplinary health research teams beyond their traditional scope and can even co-lead those teams. Further research is required to understand the experiences of trained PRPs when participating, leading or co-leading research and the impact on the research outputs.

## Data Availability

Data sharing is not applicable to this article as no datasets were generated during the current study.

## Abbreviations

CIHR: Canadian Institutes of Health Research
PRP: Patient Research Partner
IBD: Inflammatory Bowel Disease
ERA: Experience-Reflection-Action
PaCER: Patient and Community Engagement Research
IMAGINE: Inflammation, Microbiome, and Alimentation: Gastro-Intestinal and Neuropsychiatric Effects
SPOR SUPPORT: Strategy for Patient Oriented Research Support for People and Patient-Oriented Research and Trials
PCORI: Patient-Centered Outcomes Research Institute
NIHR: National Institute for Health and Care Research
